# Radiation Dose Alert: CT Overexposure As a Result of Patient Off-Centering

**DOI:** 10.5334/jbsr.3971

**Published:** 2025-12-03

**Authors:** Denis Tack

**Affiliations:** 1 Epicura Hospital, Belgium

**Keywords:** computed tomography, CT, radiation dose, automatic exposure control

## Abstract

A high cumulative radiation dose (3394 mGy.cm, 51 mSv) delivered by two acquisitions on the abdomen and pelvis and corresponding to more than 10 times the local median dose for this examination was retrospectively detected thanks to the Dose Archiving and Communicating System (DACS). Retrospective analysis of the examination showed that the cause of this high dose was an off-centering of the patient—the table being in a too high position—disabling a correct diagnosis of an umbilical herniation that was visible on a repeated acquisition. Interestingly, the CTDIvol of the second acquisition was 40% lower than that of the first acquisition, a well-known effect of off-centering on automatic exposure control systems.

In order to adapt the radiation dose to the patient absorption, CT vendors have developed automatic exposure control systems (AEC) [1]. These systems require one or two projection radiographs also named topograms or scout views to estimate the tube current necessary to warrant the required image quality. When two scout views are obtained (as with CT devices from General Electric or from Canon), the centering of the patient in the y-axis, yielded by the table height, can be achieved with high precision on the scout view obtained in the lateral orientation. When only one scout view is required by the vendor (Siemens Healthineers, Philips), the centering in the y-axis relies on the estimation of the patient center using a horizontal laser light projected on the patient. This centering is usually easy to achieve but not in obese patients who are frequently off-centered. In their technical notice to users, vendors state that an off-centering by less than 4 cm will have almost no effect on the delivered dose. If off-centering is higher than this threshold, the dose will be increased if the patient is close to the detectors during the scout view, and decreased if the patient is distant to the detectors during the scout view. The tube can be either above or below the patient during the scout view.

Figure 1 illustrates an off-centering with a table height set too high: the patient’s effective diameter is 37 cm. He is referred to CT after an inconclusive ultrasound examination of the umbilic and is suspected of umbilical herniation. After an antero-posterior topogram (first ‘topoAbdomen’ line of the dose report of Figure 1), the planned acquisition of the entire abdomen is obtained (first ‘Abdomen’ line of the dose report).

**Figure 1:**
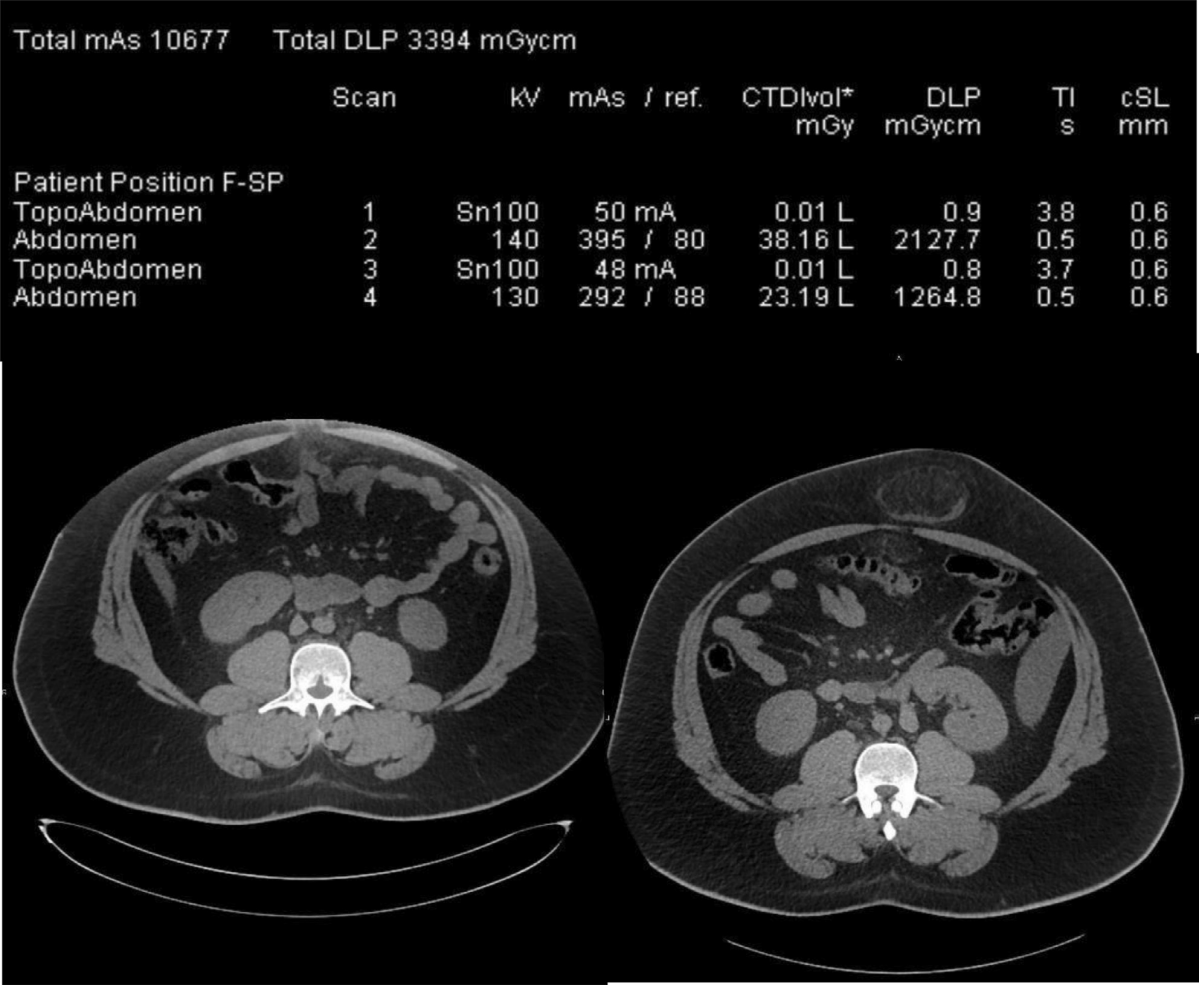
Radiation dose report and CT slices obtained at the level of the umbilic in a 24 year-old patient. The left CT slice is off-centered and the herniation is not visible whereby the right CT slice of correctly centered and the herniation is visible. The two first exposures on the dose report correspond to the left image whereby the two last lines correspond to the right image including the umbilical herniation.

Default helical acquisition parameters are the following for unenhanced abdomen–pelvis: reference KV: 120; tube potential selection is set on level 7 (of 13 positions) with ‘liver’ as corresponding symbol/organ; quality-reference mAs is set at 100. AEC strength curve is set at ‘average.’

Image quality (IQ) yielded by these acquisition parameters has been scored at 4/5 (on a subjective five-point scale, where 1 would represent ‘unacceptable’ and 5 ‘perfect’ IQ) as seen in Figure 1.

The left CT slice in Figure 1 shows that the umbilical herniation is not included in the image because it is located outside the largest available field of view. Subsequently, the radiologist asked for a repeated acquisition after the patient had been removed from the table. Therefore, the second part of the examination included both the topogram and the helical acquisitions (third and fourth exposure lines on the dose report). The patient centering was modified for the second helical acquisition and enabled to provide images that included the herniation.

The radiation dose report analysis is of interest because it shows that the anterior patient off-centering during the first acquisition induced a 40% CTDIvol increase (38.16 mGy in comparison to 23.19 mGy) as compared to the second acquisition. This dose increase can be explained by the artificial geometric increase in size of the patient placed too proximal to the detectors during the scout view and interpreted by the AEC as a larger object, thus to be scanned with an increased radiation dose [2, 3].

The total exposure delivered during the examination expressed in dose-length product (DLP) was 3394 mGy.cm, and corresponded to an effective dose of 51 mSv (conversion factor for CT of the abdomen: 0.015 mSv/mGy.cm). This is clearly an outlier and was thus depicted by our DACS (Intuitus, Telemis, Belgium) that sent an e-mail alert to the quality officer of the department.

In optimal conditions, the dose reported here above could have been reduced by

- Correctly centering the patient using a complementary lateral topogram and avoiding the first acquisition (+ 0.9 mGy.cm for the topogram and − 2227 mGy.cm for the first helical acquisitions).
- Using a reduced-dose protocol suited for urinary-stone disease and also for disorders characterized by fat infiltration such as appendicitis or diverticulitis [4–6] – 760 mGy.cm (60% of 1265 mGy.cm): 506 mGy.cm remaining. Indeed, as image quality in Figure 1 is too high for the expected diagnosis, a protocol generating higher image noise but lower dose should be used.
- Centering the z-axis landmarks in order to include the umbilic but not the entire abdomen and pelvis (–50% of the 506 remaining DLP = 253 mGy.cm)

Finally, as compared to the total delivered dose of 3394 mGy.cm, the optimized dose would have been of 253 mGy.cm + 2 topograms = 255 mGy.cm, a 13-fold reduction. This dose would have been close to the median dose value collected for this device (221 mGy.cm).

Summary: To avoid increased dose induced by AEC and due to off-centering of obese patients, a lateral topogram should be obtained and the use of lower dose protocols can be considered for this indication.
